# First‐line PD‐1/PD‐L1 inhibitors plus chemotherapy versus bevacizumab plus chemotherapy for advanced non‐squamous non‐small cell lung cancer: A Bayesian network meta‐analysis of randomized controlled trials

**DOI:** 10.1002/cam4.4589

**Published:** 2022-03-22

**Authors:** Jinzhao Zhai, Jiangyue Lu, Zhibo Zhang, Yuan Wang, Xiaoyan Li, Sujie Zhang, Shuai Mu, Xiaoyu Zhi, Xiangwei Ge, Di Lu, Yi Hu, Jinliang Wang

**Affiliations:** ^1^ Senior Department of Oncology The 5th Medical Center of Chinese PLA General Hospital Beijing China; ^2^ Medical School of Chinese PLA Beijing China; ^3^ Harbin Medical University Cancer Hospital Harbin China; ^4^ The 78th Group Army Hospital of Chinese PLA Mudanjiang China; ^5^ Haidian No. 23 Cadre's Sanitarium of Chinese PLA Beijing China

**Keywords:** bevacizumab, chemotherapy, immune checkpoint inhibitor, network meta‐analysis, non‐small cell lung cancer

## Abstract

Chemotherapy in combination with immune checkpoint inhibitor (ICI) or bevacizumab has demonstrated a superior effect for non‐squamous non‐small cell lung cancer (NS‐NSCLC). There are still few randomized controlled trials (RCTs) investigating the differences between ICI plus chemotherapy (ICI‐chemotherapy) and bevacizumab plus chemotherapy (Bev‐chemotherapy) in first‐line treatment of NS‐NSCLC. We identified RCTs in databases and conference abstracts presented at international conferences by Sep 1, 2021. Bayesian network meta‐analysis was performed using randomized effect consistency model to estimate hazard ratio (HR) and odds ratio (OR). The outcomes included overall survival (OS), progression‐free survival (PFS), overall response rate (ORR), and grade ≥ 3 treatment‐related adverse events (TRAEs). Fifteen RCTs (17 articles) of 6561 advanced NS‐NSCLC patients receiving ICI‐chemotherapy, Bev‐chemotherapy, or chemotherapy at first‐line were eligible for analysis. NMA results showed that first‐line ICI‐chemotherapy prolonged OS (HR 0.79, 0.66–0.94) in patients with advanced NS‐NSCLC compared with Bev‐chemotherapy, while no differences were in PFS, ORR, and grade ≥ 3 TRAEs (*p* > 0.05). Ranking plots suggested that ICI‐chemotherapy had the most probability to offer the best OS (probability 0.993), PFS (probability 0.658), and ORR (probability 0.565), and Bev‐chemotherapy had the most risks of grade ≥ 3 TRAEs (probability 0.833). Therefore, our findings showed that first‐line ICI‐chemotherapy was associated with better OS than Bev‐chemotherapy in patients with advanced NS‐NSCLC, and more clinical trials are warranted to confirm these results.

## Graphical abstract

ICI‐chemotherapy and bev‐chemotherapy are superior to chemotherapy alone for previous untreated NS‐NSCLC. ICI‐chemotherapy may have long‐term effect and prolong survival compared with bev‐chemotherapy without more severe TRAEs.

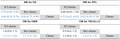

## 1 INTRODUCTION

Lung cancer remains the most common cancer and has been the main cause of cancer‐related death worldwide.^1^, ^2^ Non‐small cell lung cancer (NSCLC) accounts for more than 80% of all lung cancers, with most having non‐squamous histology.^3^ Despite significant improvements have been made in the treatment of advanced NSCLC, The prognosis remains poor with median overall survival (OS) of 8–10 months and 5‐year progression‐free survival (PFS) of 4%.^4^, ^5^ Currently, the mainstream treatment for advanced non‐squamous NSCLC (NS‐NSCLC) is to target driver gene mutation such as epidermal growth factor receptor.^6^, ^7^, ^8^ However, customarily a small fraction of patients were identified as having driver mutations that can benefit from targeted agents.^9^, ^10^ As for patients without sensitive gene mutations, they are unable to benefit from targeted therapy, making the choices of treatment full of passivity for them.^9^ Traditional chemotherapy remains the mainstream treatment with a response rate of only 15% ~ 30%.

Bevacizumab is a vascular endothelial growth factor monoclonal antibody, which inhibits angiogenesis to suppress tumor growth by restricting the delivery of oxygen and nutrients to the tumor.^11^, ^12^ Since bevacizumab was added to chemotherapy (Bev‐chemotherapy), this combination enhanced treatment efficacy and has become the standard first‐line treatment for advanced NS‐NSCLC.^13^, ^14^, ^15^, ^16^, ^17^, ^18^, ^19^, ^20^, ^21^ However, the overall survival (OS) benefit of Bev‐chemotherapy remains unsatisfactory.^16^, ^22^

The introduction of immunotherapy has a howling success, changing the treatment strategy of advanced NS‐NSCLC.^23^, ^24^, ^25^, ^26^, ^27^, ^28^, ^29^, ^30^ Immune checkpoint inhibitors (ICIs) of programmed cell death‐1/programmed cell death‐ligand 1 (PD‐1/PD‐L1) inhibitors, such as pembrolizumab, nivolumab, and atezolizumab, have been approved for advanced NS‐NSCLC because of their substantial improvements in survival compared with chemotherapy.^31^, ^32^, ^33^, ^34^ Recently, the IMpower‐150 trial showed that first‐line ICI plus chemotherapy (ICI‐chemotherapy) had survival benefit compared with Bev‐chemotherapy in patients with NS‐NSCLC.^35^, ^36^ For lack of head‐to‐head comparison, the safety, efficacy, and prognosis of both combination strategies in first‐line treatment remain uncertain. Therefore, we carried out this network meta‐analysis (NMA) to determine whether ICI‐chemotherapy prolongs survival in advanced untreated NS‐NSCLC compared to Bev‐chemotherapy.

## 2 METHODS

### 2.1 Data sources and search strategy

We conducted a systematic search from PubMed, Embase, and the Cochrane library up to Sep 1, 2021. We also searched the conference abstracts published at international conferences to identify eligible trials. The search was conducted under the principles outlined in the Cochrane Handbook for Systematic Reviews of Interventions. The search strategies were as follows: (“PD‐L1” OR “programmed cell death‐1” OR “PD‐1” OR “nivolumab” OR “opdivo” OR “pembrolizumab” OR “keytruda” OR “atezolizumab” OR “tecentriq” OR “durvalumab” OR “Imfinzi” OR “avelumab”) OR (“bevacizumab” OR “avastin”) AND (“non‐small cell lung” OR “nsclc” OR “lung adenocarcinoma” OR “nonsquamous”) AND (“advanced” OR “metastatic”) AND (“chemotherapy” OR “carboplatin” OR “cisplatin” OR “paclitaxel” OR “docetaxel” OR “pemetrexed”) AND (“first‐line” OR “untreated” OR “treatment naïve” OR “chemo naïve” OR “front line”) AND (“controlled clinical trial” OR “RCT” OR “randomly” OR “trial”). The detailed strategies are shown in Supplement 1.

### 2.2 Inclusion and exclusion criteria

Studies were included as follows: (1) patients had histologically confirmed with advanced NS‐NSCLC (stage IIIB‐IV); (2) studies involved the comparisons among ICI‐chemotherapy, Bev‐chemotherapy, and chemotherapy in first‐line treatment; (3) study outcomes included objective response rate (ORR), PFS, OS, or grade ≥ 3 treatment‐related adverse events (TRAEs); (4) study designs were controlled clinical trials (RCTs). Studies were excluded as follows: (1) studies with duplicate publications; (2) study protocols, reviews, meta‐analyses, letters, or case reports; (3) studies with data unavailable. If the trial compared one drug of two different dosages, we chose the usual drug dosage. This NMA was conducted following the Preferred Reporting Items for Systematic Reviews and Meta‐Analyses (PRISMA).^37^

### 2.3 Outcome measures

The following outcomes were used for analysis: ORR, PFS, OS, and grade ≥ 3 TRAEs. ORR was calculated by the proportion of complete and partial recession. PFS was defined as the time between randomization and disease progression or death from any cause. OS was calculated as the time in between randomization and death from any cause. Grade ≥ 3 TRAEs were evaluated by the common terminology criteria for adverse events.^38^ Hazard ratio (HR) with 95% confidence interval (CI) was calculated for continuous variable analysis (OS and PFS), and odds ratio (OR) with 95%CI were estimated for binary variable analysis (ORR and grade ≥ 3 TRAEs).

### 2.4 Study selection and data extraction

Two investigators (Z.B. Zhang and Y. Wang) independently screened the studies by title, abstract, and full text based on inclusion and exclusion criteria; any discrepancies were discussed by a third reviewer (J.L. Wang). The extracted data included the first author, publication year, trial name, trial phase, sex, age, smoking history, treatment strategy, drug dose, number of patients, Eastern Cooperative Oncology Group Performance Status (ECOG PS), and the outcomes of OS, PFS, ORR, and grade ≥ 3 TRAEs.

### 2.5 Quality assessment

The quality of each included study was assessed by another two reviewers (J.Y. Lu and X.W. Ge) independently with the Cochrane Risk of Bias Tools,^39^ including six domains: (1) randomization sequence generation and allocation concealment; (2) blinding of patients and personnel; (3) blinding of outcome assessment; (4) incomplete outcome data; (5) selective outcome reporting; (6) other bias. Discrepancies were mediated by a third investigator (J.L. Wang) and resolved by consensus. Quality assessments for each trial are available in Supplement 2.

### 2.6 Statistical analysis

#### 2.6.1 Pairwise meta‐analysis

STATA software (version 15.0) was used for performing pairwise meta‐analysis (PMA) in a random‐effect model. The *I*‐square was calculated for assessing the extent of variability attributable to heterogeneity between two studies.^40^ PMA between ICI‐chemotherapy and chemotherapy was conducted stratified by PD‐L1 expression, liver metastases, and brain metastases.

#### 2.6.2 Network meta‐analysis

R software (version 4.1.0) with gemtc package (version 1.0) was applied for conducting Bayesian network meta‐analysis (NMA) in a random‐effect model. Four chains were generated and 100,000 iterations with 10,000 burn‐ins for each chain (the interval of 10). R software was also used for identifying the probability of each treatment to be ranked the best for four endpoints and the surface under the cumulative ranking curves (SUCRAs) were presented in ranking plots. Network plots showed the connection between eligible trails based on the number of trails and sample size. The comparison‐adjusted funnel plots tested publication bias.^41^, ^42^ To ensure the reliability of NMA, sensitive analysis was conducted by excluding 1 trial with phase II and 3 trials with a sample size of each group less than 100. Inconsistency was assessed by comparing the synthesized HRs of PMA and NMA results. Node splitting analysis was used for assessing the consistency of direct and indirect evidence.^43^ The contribution plot was used to measure the percent contribution of direct comparison to the indirect, mixed, and the entire network estimates (Supplementary materials). All tests were two‐sided, and a *p*‐value below 0.05 was considered statistically significant.

## 3 RESULTS

### 3.1 Eligible studies and characteristics

**FIGURE 1:**
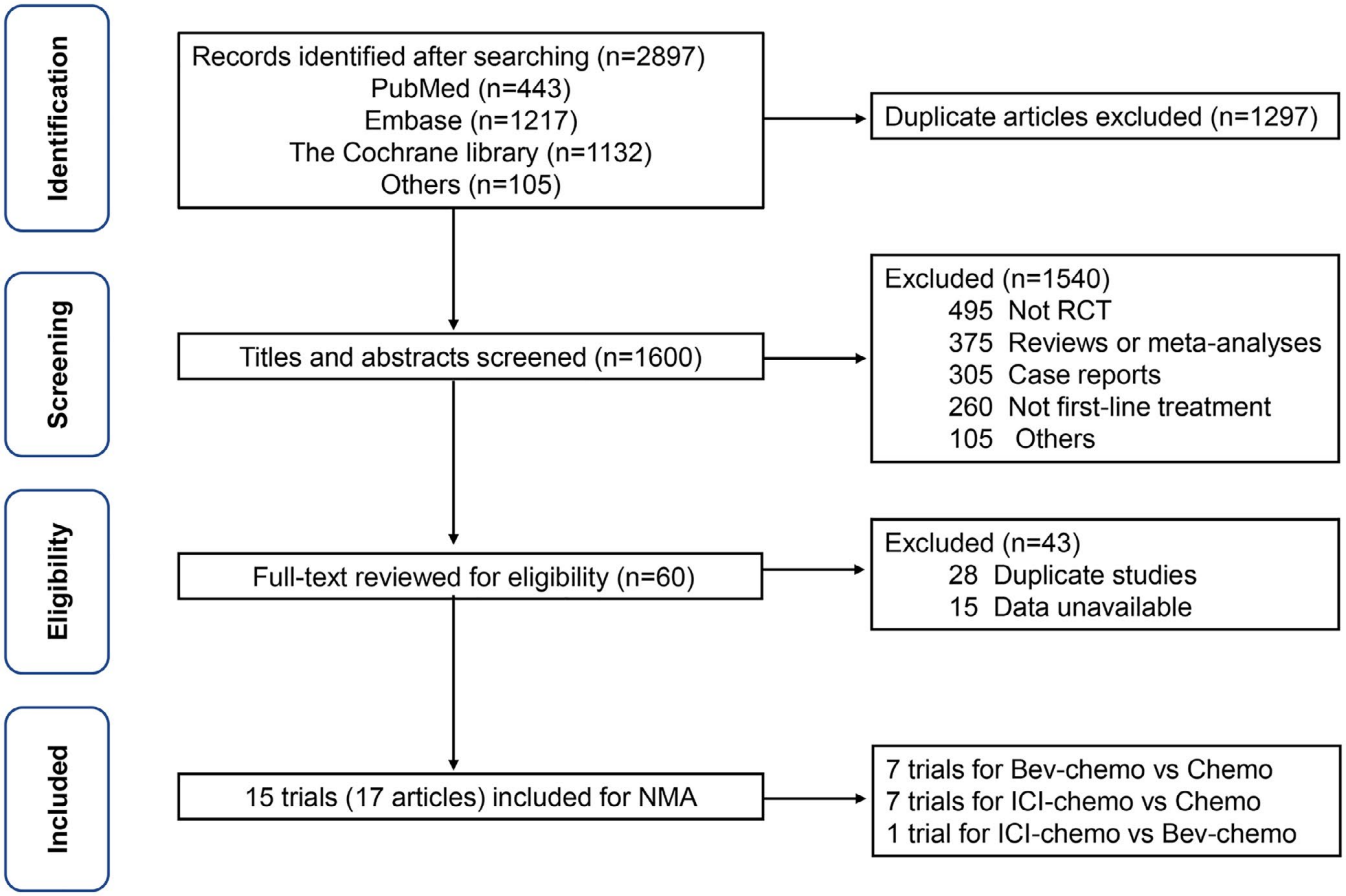
Flowchart of eligible studies selection

**FIGURE 2:**
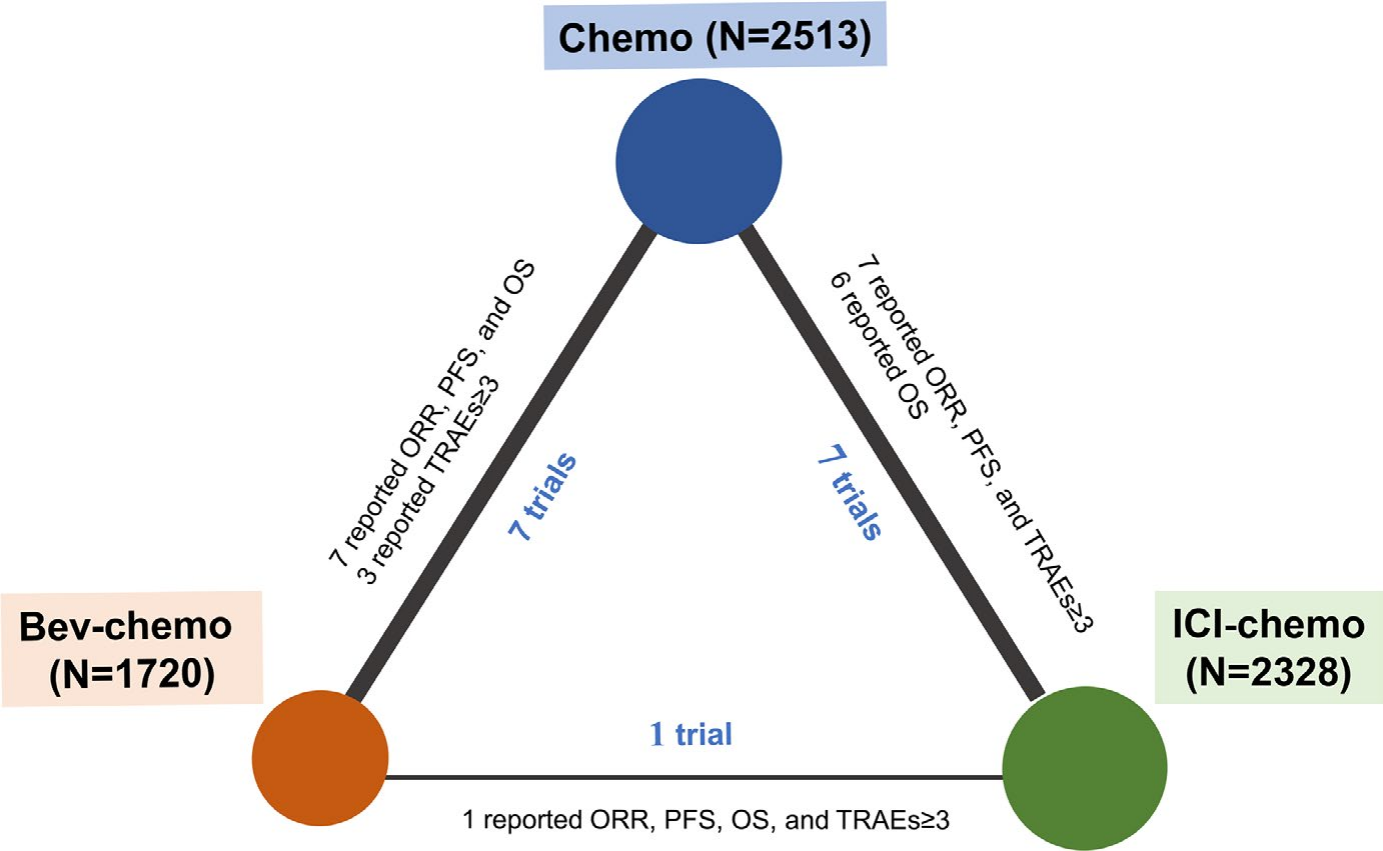
Network plot of all eligible trials. The area of the circles means the proportional number of patients for each treatment. The thickness of the lines means the proportional number of trials comparing the connected two treatments. PFS, progression‐free survival; OS, overall survival; ORR, objective response rate; TRAEs, treatment‐related adverse events; ICI, immune checkpoint inhibitor; bev, bevacizumab; chemo, chemotherapy

All 2284 records were identified from the databases and international conferences. After screening titles and abstracts to exclude duplicate articles and articles that did not meet the inclusion criteria, 15 eligible RCTs (17 articles) with 6541 advanced NS‐NSCLC patients receiving three treatments (ICI‐chemotherapy, Bev‐chemotherapy, and chemotherapy) were included in this NMA. Seven trials^15^, ^16^, ^17^, ^18^, ^19^, ^20^, ^21^ compared Bev‐chemotherapy with chemotherapy, seven trials^23^, ^24^, ^25^, ^26^, ^27^, ^28^, ^29^, ^30^ compared ICI‐chemotherapy with chemotherapy, and one trial^35^, ^36^ compared ICI‐chemotherapy with Bev‐chemotherapy (Figure 1). Overall, ICI‐chemotherapy, Bev‐chemotherapy, and chemotherapy were administered to 2328, 1720, and 2513 patients, respectively. Network plots of all eligible trials were shown in Figure 2. Of all 15 trials, 15 reported ORR and PFS, 14 reported OS, and 11 reported grade ≥ 3 TRAEs. Thirteen of 15 trials (86.7%) were phase III RCTs; 12 of 15 RCTs (80%) included more than 100 patients in each arm. Eligible studies and patients' characteristics (Table 1 and Table 2) included the first author, publication year, trial name, trial phase, treatment drug, number of patients, sex, age, stage, smoking status, ECOG PS, brain metastases, and liver metastases. Supplement 2 showed that 20.0% (3/15) were high‐quality studies and 46.7% (7/15) were accompanied by a high risk of bias for lacking personnel blinding according to the Cochrane Collaboration tool.

**TABLE 1:** Characteristics of included studies in network meta‐analysis

Author (year) Trial	Experimental Arm (E)	Control Arm (C)	Phase	Histology	Patients	PFS	OS	ORR (%)	Grade ≥ 3 TRAEs (%)
					E/C	HR (95%CI)	HR (95%CI)	E/C	E/C
Bev‐chemo versus Chemo									
Sandler et al. (2006) ECOG4599^15^	Bev + Paclitaxel + Carboplatin	Paclitaxel + Carboplatin	III	NS‐NSCLC	417/433	0.66 (0.57, 0.77)	0.79 (0.67, 0.92)	34.9/15.1	60.9/24.3
Reck et al. (2010) AVAiL^16^	Bev + Gemcitabine + Cisplatin	Gemcitabine + Cisplatin + Placebo	III	NS‐NSCLC	351/347	0.82 (0.68, 0.98)	1.03 (0.86, 1.23)	30.4/20.1	80.5/75.2
Niho et al. (2012) JO19907^17^	Bev + Gemcitabine + Cisplatin	Gemcitabine + Cisplatin	II	NS‐NSCLC	121/59	0.61 (0.42, 0.89)	0.99 (0.65, 1.50)	60.7/31.0	NA
Boutsikou et al. (2013)^18^	Bev + Docetaxel + Carboplatin	Docetaxel + Carboplatin	III	NS‐NSCLC	56/61	0.37 (0.32, 0.51)	0.77 (0.38, 1.6)	39/31	NA
Galetta et al. (2015) ERACLE^19^	Bev + Paclitaxel + Carboplatin	Pemetrexed + Cisplatin	III	NS‐NSCLC	58/60	0.79 (0.53, 1.17)	0.93 (0.6, 1.42)	51.7/40.0	NA
Zinner et al. (2015) PRONOUNCE^20^	Bev + Paclitaxel + Carboplatin	Pemetrexed + Carboplatin	III	NS‐NSCLC	179/182	0.94 (0.74, 1.19)	0.93 (0.74, 1.20)	27.4/23.6	NA
Zhou et al. (2015) BEYOND^21^	Bev + Paclitaxel + Carboplatin	Paclitaxel + Carboplatin + Placebo	III	NS‐NSCLC	138/138	0.40 (0.29, 0.54)	0.68 (0.50, 0.93)	53.6/25.4	67.1/61.9
ICI‐chemo versus Chemo									
West et al. (2019) IMpower‐130^23^	Atezo + Nabpaclitaxel + Carboplatin	Nabpaclitaxel + Carboplatin	III	NS‐NSCLC without EGFR or ALK mutation	483/241	0.64 (0.54,0.77)	0.79 (0.64,0.98)	49.2/31.9	74.8/60.8
Gadgeel et al. (2020) KEYNOTE‐189^26^	Pembro + Pemetrexed + Cisplatin	Pemetrexed + Cisplatin	III	NS‐NSCLC without EGFR or ALK mutation	410/206	0.48 (0.40, 0.58)	0.56 (0.45, 0.70)	47.6/18.9	71.9/66.8
Zhou et al. (2021) CameL^24^, ^30^	Camrelizumab + Pemetrexed + Carboplatin	Pemetrexed + Carboplatin + Placebo	III	NS‐NSCLC without EGFR or ALK mutation	193/196	0.37 (0.29, 0.47)	0.55 (0.40, 0.75)	64.8/36.7	73.6/71.9
Awad et al. (2021) KEYNOTE‐021^25^	Pembro + Pemetrexed + Carboplatin	Pemetrexed + Carboplatin	II	NS‐NSCLC without EGFR or ALK mutation	60/63	0.54 (0.35, 0.83)	0.71 (0.45, 1.12)	63.6/40.0	39.0/30.6
Lu et al. (2021) RATIONALE‐304^27^	Tislelizumab + Platinum + Pemetrexed	Platinum + Pemetrexed	III	NS‐NSCLCwithout EGFR or ALK mutation	222/110	0.56 (0.41, 0.77)	NA	57.4/36.9	67.6/53.6
Nishio et al. (2021) IMpower‐132^28^	Atezo + Pemetrexed + Carboplatin or Cisplatin	Pemetrexed + Carboplatin or Cisplatin	III	NS‐NSCLC without EGFR or ALK mutation	292/286	0.60 (0.49, 0.72)	0.81 (0.64, 1.03)	47/32	54.6/40.1
Yang et al. (2021) ORIENT‐11^29^	Sintilimab + Pemetrexed + Platinum	Pemetrexed + Platinum + Placebo	III	NS‐NSCLC without EGFR or ALK mutation	266/131	0.49 (0.38, 0.63)	0.60 (0.45, 0.79)	51.9/29.8	61.7/58.8
ICI‐chemo versus Bev‐chemo									
Socinski et al. (2021) IMpower‐150^35^	Atezo + Paclitaxel + Carboplatin	Bev + Carboplatin + Paclitaxel	III	NS‐NSCLC without EGFR or ALK mutation	402/400	0.84 (0.71, 1.00)	0.86 (0.73, 1.01)	40.6/40.2	59.0/63.7

ABBREVIATIONS: atezo, atezolizumab; bev, bevacizumab; chemo, chemotherapy; HR, hazard ratio; ICI, immune checkpoint inhibitor; NA, not available; NS‐NSCLC, non‐squamous non‐small cell lung cancer; ORR, objective response rate; OS, overall survival; pembro, pembrolizumab; PFS, progression‐free survival.

**TABLE 2:** Patients' characteristics of included studies

No.	Trial	Experimental Arm (E)	Control Arm (C)	E/C (%)							
				Patients	Male	Age (median)	Stage IV	Smokers	ECOG = 0	Brain metastases	Liver metastases
1	ECOG4599	Bev‐chemo	Chemo	417/433	50/58	56/58	74/78	——	40/40	——	22/17
2	AVAiL	Bev‐chemo	Chemo	351/347	62/64	59/59	77/77	——	41/41	——	——
3	JO19907	Bev‐chemo	Chemo	121/59	64/64	56/57	69/71	69/68	51/49	——	——
4	Boutsikou 2013	Bev‐chemo	Chemo	56/61	80/85	63/65	73/84	84/87	——	——	——
5	ERACLE	Bev‐chemo	Chemo	58/60	78/70	62/60	93/95	60/70	79/78	——	——
6	BEYOND	Bev‐chemo	Chemo	138/138	54/56	57/56	91/91	50/44	25/20	——	——
7	PRONOUNCE	Bev‐chemo	Chemo	179/182	58/58	65/66	100/99.5	96/90	46.9/46.7	12.6/17.9	——
8	IMpower‐132	ICI‐chemo	Chemo	292/286	66/67	64/63	100/100	87.3/89.5	43.2/40.1	——	12.7/12.6
9	IMpower‐130	ICI‐chemo	Chemo	483/241	59/59	64/65	100/100	89/93	42/40	——	15 /14
10	CameL	ICI‐chemo	Chemo	193/196	71/72	59/61	85/80	62/63	23/17	5/2	——
11	RATIONALE‐304	ICI‐chemo	Chemo	222/110	75.3/71.2	60/61	82.1/81.1	65.9/59.4	24.2/21.6	4.9/6.3	9.0/15.3
12	KEYNOTE‐021	ICI‐chemo	Chemo	60/63	37/41	63/66	98/95	75/86	40/46	20/11	——
13	ORIENT‐11	ICI‐chemo	Chemo	266/131	76.7/75.6	61/61	92.1/88.5	64/66	29/26	13.5/16.8	——
14	KEYNOTE‐189	ICI‐chemo	Chemo	410/206	62/53	65/64	100/100	88/88	45/39	17.8/17.0	16.1/23.8
15	IMpower‐150	ICI‐chemo	Bev‐chemo	402/400	60/60	63/63	100 /100	81/81	44.8/40.1	——	13/14

### 3.2 NMA of outcomes

As listed in Table 3, four outcomes were evaluated by NMA. For OS, the pooled results showed that ICI‐chemotherapy (HR 0.68, 95% CI: 0.59–0.78) or Bev‐chemotherapy (HR 0.86, 95% CI: 0.75–0.99) improved OS compared with chemotherapy. ICI‐chemotherapy was associated with better OS than Bev‐chemotherapy (HR 0.79, 95%CI: 0.66–0.94). For PFS, the addition of ICI (HR 0.52, 95% CI: 0.42–0.65) or bevacizumab (HR 0.62, 95%CI: 0.50–0.78) to chemotherapy decreased the risk of disease progression, but there were no statistical differences in PFS between ICI‐chemotherapy and Bev‐chemotherapy (*p* > 0.05). For ORR, patients with either ICI‐chemotherapy (OR 1.7, 95% CI: 1.5–2.0) or Bev‐chemotherapy (OR 1.7, 95% CI: 1.4–2.0) had higher ORR than those with chemotherapy, while there were no significant differences in two combination therapies (*p* > 0.05). For grade ≥ 3 TRAEs, patients with Bev‐chemotherapy (OR 1.4, 95% CI: 1.0–1.9) had more grade ≥ 3 TRAEs than chemotherapy, while no statistical differences were identified between ICI‐chemotherapy and Bev‐chemotherapy (*p* > 0.05).

**TABLE 3:** Network meta‐analysis of clinical outcomes

HR for OS			HR for PFS		
ICI‐chemo			ICI‐chemo		
0.79 (0.66, 0.94)	Bev‐chemo		0.84 (0.63, 1.10)	Bev‐chemo	
0.68 (0.59, 0.78)	0.86 (0.75, 0.99)	Chemo	0.52 (0.42, 0.65)	0.62 (0.50, 0.78)	Chemo
**OR for ORR**			**OR for TRAEs ≥ 3**		
ICI‐chemo			ICI‐chemo		
1.0 (0.8, 1.3)	Bev‐chemo		0.9 (0.6, 1.2)	Bev‐chemo	
1.7 (1.5, 2.0)	1.7 (1.4, 2.0)	Chemo	1.2 (0.9, 1.5)	1.4 (1.0, 1.9)	Chemo

Abbreviations: bev, bevacizumab; chemo, chemotherapy; HR, hazard ratio; ICI, immune checkpoint inhibitor; OR, odds ratio; objective response rate; OS, overall survival; PFS, progression‐free survival; TRAEs, treatment‐related adverse events.

### 3.3 Treatment ranking

**FIGURE 3:**
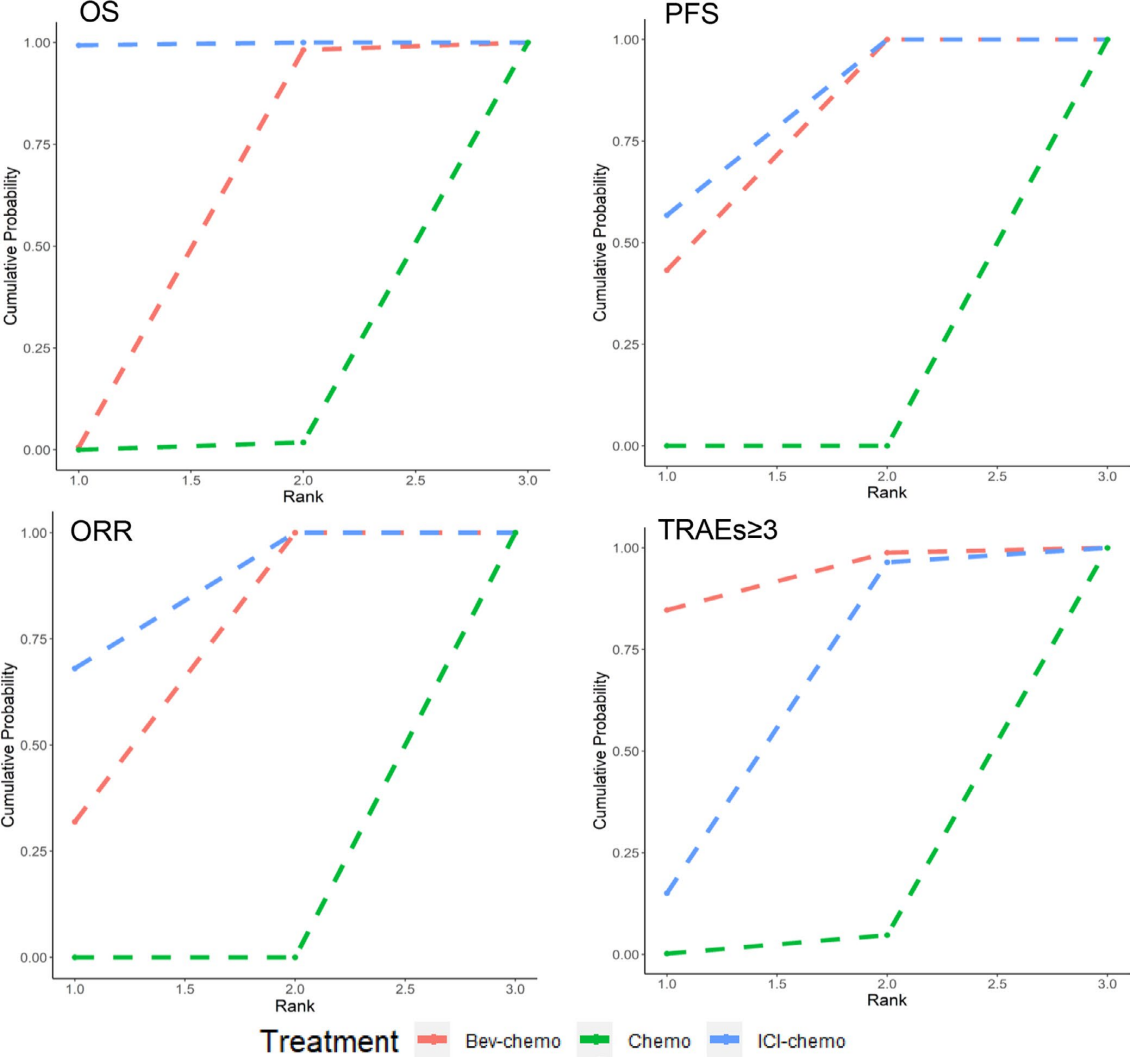
Treatment ranking plot

The treatment ranking plots for four outcomes were shown in Figure 3, which suggested that ICI‐chemotherapy had the most probability to offer the best OS (probability 0.993), PFS (probability 0.658), ORR (probability 0.565), and Bev‐chemotherapy had the most risks of grade ≥ 3 TRAEs (probability 0.833).

### 3.4 Subgroup analysis

**FIGURE 4:**
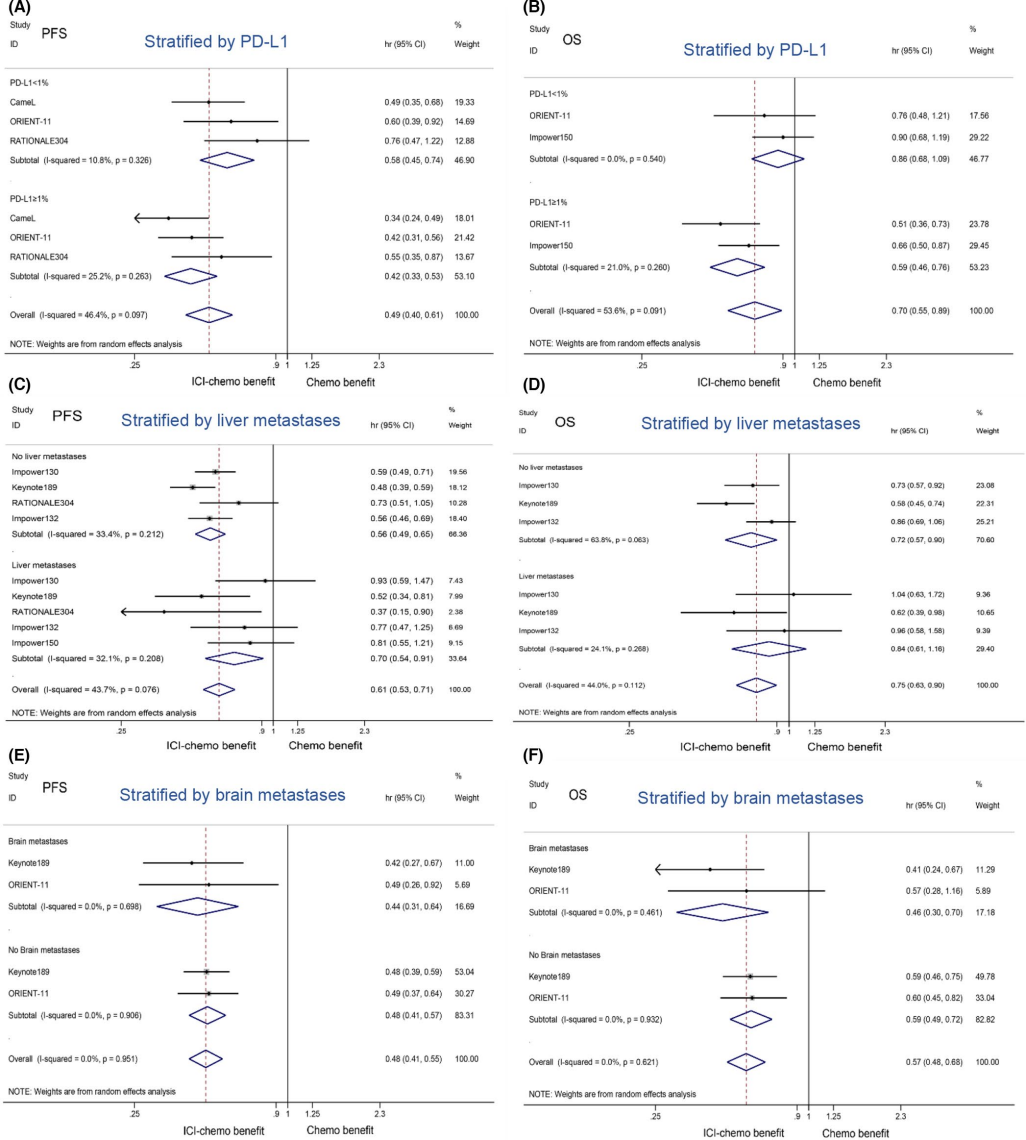
Comparing ICI‐chemotherapy with chemotherapy stratified by PD‐L1 expression, liver metastases, and brain metastases

Subgroup PMA was conducted for comparing ICI‐chemotherapy with chemotherapy. Stratified by PD‐L1 expression, patients with PD‐L1 ≥ 1% (HR 0.42, 95%CI: 0.33–0.53) or PD‐L1 < 1% (HR 0.58, 95%CI: 0.45–0.74) had PFS benefit, while only patients with PD‐L1 ≥ 1% (HR 0.59, 95%CI: 0.46–0.76) had OS benefit. Stratified by liver metastases, ICI‐chemotherapy was associated with longer PFS in patients with liver metastases (HR 0.70, 95%CI: 0.54–0.91) or no liver metastases (HR 0.56, 95%CI: 0.49–0.65) compared with chemotherapy, while OS benefit only existed in patients with no liver metastases (HR 0.72, 95%CI: 0.57–0.90). Stratified by brain metastases, ICI‐chemotherapy was in association with better PFS and OS for patients with no brain metastases (PFS: HR 0.44, 95%CI: 0.31–0.64; OS: HR 0.46, 95%CI: 0.30–0.70) or brain metastases (PFS: HR 0.48, 95%CI: 0.41–0.57; OS: HR 0.59, 95%CI: 0.49–0.72) than chemotherapy (Figure 4).

Subgroup NMA was performed in patients with liver metastases, including one trial comparing ICI‐chemotherapy with Bev‐chemotherapy, three trials comparing ICI‐chemotherapy with chemotherapy, and one trial comparing Bev‐chemotherapy with chemotherapy (**Supplement 3**). The results demonstrated that there were no significant differences in PFS and OS among three treatments (ICI‐chemotherapy, Bev‐chemotherapy, and chemotherapy) in patients with liver metastases (*P* > 0.05) (Supplement 4).

### 3.5 Heterogeneity and publication bias

Heterogeneity was evaluated in direct, indirect, and network comparations. The results showed that there was little heterogeneity among included trials with *I* ^2^ > 50% (Supplement 5). Comparison‐adjusted funnel plots were presented in Supplement 6, which indicated little publication bias among included trials.

### 3.6 Inconsistency assessment

**FIGURE 5:**
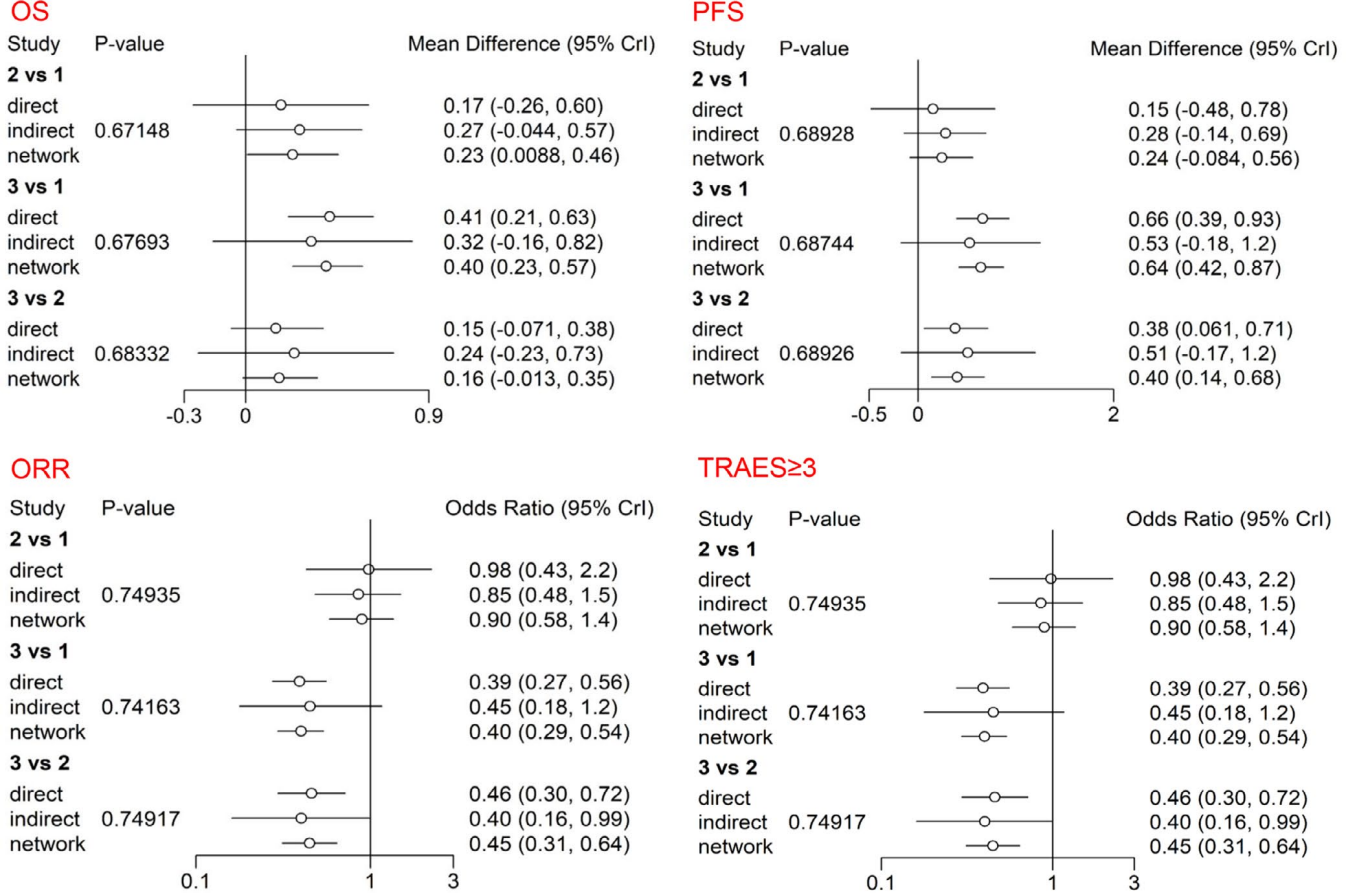
Forest plots for direct, indirect, and network meta‐analysis (NMA). 1: ICI‐chemotherapy, 2: Bev‐chemotherapy, 3: Chemotherapy. PFS, progression‐free survival; OS, overall survival; ORR, objective response rate; TRAEs, treatment‐related adverse events; ICI, immune checkpoint inhibitor; bev, bevacizumab; chemo, chemotherapy

The inconsistency assessment was performed using node‐splitting analysis for OS, PFS, ORR, and grade ≥ 3 TRAEs, the results showed the consistency among direct, indirect, and network comparations with all *p* > 0.05. Forest plots of direct, indirect, and network comparations were generated for OS, PFS, ORR, and grade ≥ 3 TRAEs (Figure 5).

### 3.7 Sensitivity analysis and contribution of direct comparison

As shown in Supplement 7, we excluded one trial^17^ with phase II and three trials^18^, ^19^, ^25^ with a sample size of less than 100 in each group. A total of 11 trials, involving 6023 previous untreated NS‐NSCLC patients, were enrolled in the sensitivity analysis. The results were similar to the main analysis. Therefore, we included all trials for the robustness of the final NMA results. The contribution plots of direct comparison are presented in Supplement 8.

## 4 DISCUSSION

Immune checkpoint inhibitors (ICIs) have been noted to show promise for cancer treatment due to their advances in cancer treatments and have been clinically approved in a variety of malignancies.^44^, ^45^, ^46^ PD‐1, an essential regulator of adaptive immune responses, is primarily involved in immune inhibitory signaling and is ectopically expressed on antitumor T cells, while expression of its ligand PD‐L1 can be upregulated by tumor cells and block antitumor effects.^47^, ^48^ PD‐1/PD‐L1 inhibitors could abolish this suppression effect and reactivate the antitumor effect of T cells. Blocking the PD‐1/PD‐L1 pathway has emerged as a front‐line treatment strategy for various cancers, in particular NSCLC. Unlike chemotherapy, blocking immune checkpoint could indirectly target tumors by boosting antitumor immune responses and these effects have been reported to be durable in a subset of patients.^48^

Conventional chemotherapy is the main treatment option for NS‐NSCLC patients.^49^ The combination of ICIs and chemotherapy have higher efficacy compared to chemotherapy and is therefore recommended by NCCN guidelines as first‐line treatment of advanced NS‐NSCLC.^50^, ^51^ Currently, ICI‐chemotherapy has emerged as a new treatment option on advanced NS‐NSCLC patients without the driver gene mutation. Bevacizumab, a monoclonal antibody against VEGFR, is beneficial in combination with chemotherapy for advanced NS‐NSCLC, and Bev‐chemotherapy has been the standard treatment for advanced NS‐NSCLC. IMpower‐150 trial was the first RCT comparing ICI‐chemotherapy with Bev‐chemotherapy in previous untreated NS‐NSCLC patients with advanced‐stage, the results showed that patients with ICI‐chemotherapy had prolonged survival compared with those with Bev‐chemotherapy.^35^ The comparison between first‐line ICI‐chemotherapy and Bev‐chemotherapy in advanced NS‐NSCLC remains unclear due to the lack of direct comparisons between ICI‐chemotherapy and Bev‐chemotherapy. Therefore, we performed this NMA to compare the potential of these two combination strategies.

We initially performed NMA among ICI‐chemotherapy, Bev‐chemotherapy, and chemotherapy for previously untreated patients with advanced NS‐NSCLC. Based on 15 RCTs, the results indicated an OS benefit with ICI‐chemotherapy compared to Bev‐chemotherapy, while no differences were observed on ORR, PFS, and grade ≥ 3 TRAEs. Treatment ranking plots revealed that ICI‐chemotherapy was most likely to deliver the best OS, PFS, and highest risk of TRAEs ≥3, while Bev‐chemotherapy had the highest ORR. Furthermore, we performed subgroup NMA in patients with liver metastases, and no differences in PFS and OS were detected between ICI‐chemotherapy and Bev‐chemotherapy. Our findings demonstrated that first‐line ICI‐chemotherapy was associated with better OS than Bev‐chemotherapy in advanced EC patients except for those with liver metastases. A comparison between ICI‐chemotherapy and Bev‐chemotherapy stratified by biomarkers such as PD‐L1 expression was not performed due to the unavailability of data, which requires further investigation.

There were several limitations in this study. First, different PD‐1/PD‐L1 inhibitors (pembrolizumab, camrelizumab, tislelizumab, and sintilimab) and chemotherapy regimens were used in different RCTs, which may have affected the final results. Second, the genetic status in Bev‐chemotherapy trials was unknown, whereas immunotherapy trials involved patients without the driver gene mutation. Third, some trials allowed patients to cross over from chemotherapy to ICI‐chemotherapy after progressions such as the KEYNOTE‐021 trial and CameL trial, which may decrease the OS benefit of ICI‐chemotherapy. Fourth, heterogeneity and publication bias existed, several trials are still ongoing with incomplete data, we thus conducted sensitive analysis and the results were consistent with the main analysis. Lastly, due to the unavailability of data, we only performed a subgroup NMA stratified by liver metastases. Despite the above limitations, our study still confirmed the favorable survival of ICI‐chemotherapy versus Bev‐chemotherapy without more severe TRAEs.

## 5 CONCLUSION

This study elucidates that ICI‐chemotherapy is superior to Bev‐chemotherapy for improved OS in first‐line treatment of advanced NS‐NSCLC. More clinical trials are warranted to confirm these results.

## CONFLICT OF INTEREST

All authors declare that there is no conflict of interest.

## AUTHOR CONTRIBUTIONS

J.L. Wang and Y. Hu conceived and designed the research. Data collection and extraction were performed by Z.B. Zhang and verified by Y. Wang. Statistical analysis was performed by Z.B. Zhang and Y. Wang. All authors participated in drafting and revising this article and gave final approval to the version submitted.

## Supporting information


Supinfo

## Data Availability

Data sharing is not applicable to this article as no new data were created or analyzed in this study.
