# Supplementary material for: First‐line PD‐1/PD‐L1 inhibitors plus chemotherapy versus bevacizumab plus chemotherapy for advanced non‐squamous non‐small cell lung cancer: A Bayesian network meta‐analysis of randomized controlled trials
Source: Cancer Med. 2022 Mar 22;11(10):2043–55. doi: 10.1002/cam4.4589 (PMC9119358; doi:10.1002/cam4.4589)
Supplement: Supplementary file 1 — Supinfo [file CAM4-11-2043-s001.docx]

**Supplementary materials**

1. Search strategy
2. Quality evaluation of included trails
3. Network plot of patients with liver metastases
4. Network meta-analysis of patients with liver metastases
5. Heterogeneity evaluation
6. Funnel plots for OS, PFS, ORR, and grade ≥ 3 TRAEs
7. Sensitive analysis excluding trials with phase II and trails with a sample size of each group below 100
8. Contribution plots for OS, PFS, ORR, and grade ≥ 3 TRAEs

**Supplement 1. Search Strategy**

**The searching strategy in PubMed**

| **No.** | **Query** | **Results** |
| --- | --- | --- |
| #1 | "programmed cell death-1" OR "programmed cell death ligand-1" OR PD-1 OR PD-L1 OR nivolumab OR opdivo OR pembrolizumab OR keytruda OR atezolizumab OR tecentriq OR durvalumab OR imfinzi OR avelumab | 38066 |
| #2 | carcinoma, non-small cell lung [MeSH] OR nsclc OR nonsquamous* OR lung adenocarcinoma | 124379 |
| #3 | advanced OR metastatic | 2268974 |
| #4 | bevacizumab OR avastin | 20400 |
| #5 | chemotherapy OR carboplatin OR cisplatin OR paclitaxel OR docetaxel OR pemetrexed | 3665627 |
| #6 | "first-line" OR "untreated" OR "treatment naive" OR "chemo naive" OR "front line" | 293137 |
| #7 | randomized controlled trial[Title/Abstract] OR RCT[Title/Abstract] OR controlled clinical trial[Title/Abstract] OR randomized[Title/Abstract] OR randomly [Title/Abstract] OR trial [Title/Abstract] | 1270593 |
| #8 | #1 OR #4 | 57861 |
| #9 | #2 AND #3 AND #5 AND #6 AND #7 AND #8 | 469 |
| #10 | "2005/01/01"[Date - Publication] : "2021/09/01"[Date - Publication] | 16735509 |
| #11 | #9 AND #10 | 443 |

**The searching strategy in Embase**

| No. | Query | Results |
| --- | --- | --- |
| #1 | 'programmed cell death-1' OR 'programmed cell death ligand-1' OR 'pd 1'/exp OR 'pd 1' OR 'pd l1' OR 'nivolumab'/exp OR nivolumab OR 'opdivo'/exp OR opdivo OR 'pembrolizumab'/exp OR pembrolizumab OR 'keytruda'/exp OR keytruda OR 'atezolizumab'/exp OR atezolizumab OR 'tecentriq'/exp OR tecentriq OR 'durvalumab'/exp OR durvalumab OR 'imfinzi'/exp OR imfinzi OR 'avelumab'/exp OR avelumab | 85409 |
| #2 | 'non-small cell lung cancer'/exp OR 'nsclc':ab,ti OR 'nonsquamous':ab,ti OR 'adenocarcinoma':ab,ti | 365679 |
| #3 | 'advanced':ab,ti OR 'metastatic':ab,ti | 1041817 |
| #4 | 'bevacizumab':ab,ti OR 'avastin':ab,ti | 32676 |
| #5 | 'chemotherapy':ab,ti OR 'carboplatin':ab,ti OR 'cisplatin':ab,ti OR 'paclitaxel':ab,ti OR 'docetaxel':ab,ti OR 'pemetrexed':ab,ti | 720926 |
| #6 | 'first-line':ab,ti OR 'treatment naive':ab,ti OR 'chemo naive':ab,ti OR 'front line':ab,ti | 196532 |
| #7 | 'randomized controlled trial':ab,ti OR 'rct':ab,ti OR 'controlled clinical trial':ab,ti OR 'randomized':ab,ti OR 'randomly':ab,ti OR 'trial':ab,ti | 1803936 |
| #8 | #1 OR #4 | 116592 |
| #9 | #2 AND #3 AND #5 AND #6 AND #7 AND #8 AND [1-1-2005]/sd NOT [2-9-2021]/sd AND [2005-2021]/py | 1217 |

**The searching strategy in Cochrane Library**

| No. | Query | Results |
| --- | --- | --- |
| #1 | "programmed cell death-1" OR "programmed cell death ligand-1" OR PD-1 OR PD-L1 OR nivolumab OR opdivo OR pembrolizumab OR keytruda OR atezolizumab OR tecentriq OR durvalumab OR imfinzi OR avelumab | 6980 |
| #2 | non small cell* OR non-small cell* OR nsclc OR nonsquamous* OR adenocarcinoma | 33709 |
| #3 | advanced OR metastatic | 84405 |
| #4 | bevacizumab OR avastin | 6864 |
| #5 | chemotherapy OR carboplatin OR cisplatin OR paclitaxel OR docetaxel OR pemetrexed | 97272 |
| #6 | "first-line" OR untreated OR "treatment naive" OR "chemo naive" OR "front line" | 48193 |
| #7 | "randomized controlled trial" OR RCT OR "controlled clinical trial" OR randomized OR randomly OR trial | 1518224 |
| #8 | #1 OR #4 | 13377 |
| #9 | #2 AND #3 AND #5 AND #6 AND #7 AND #8 with Cochrane Library publication date Between Jan 2005 and Sep 2021 | 1132 |


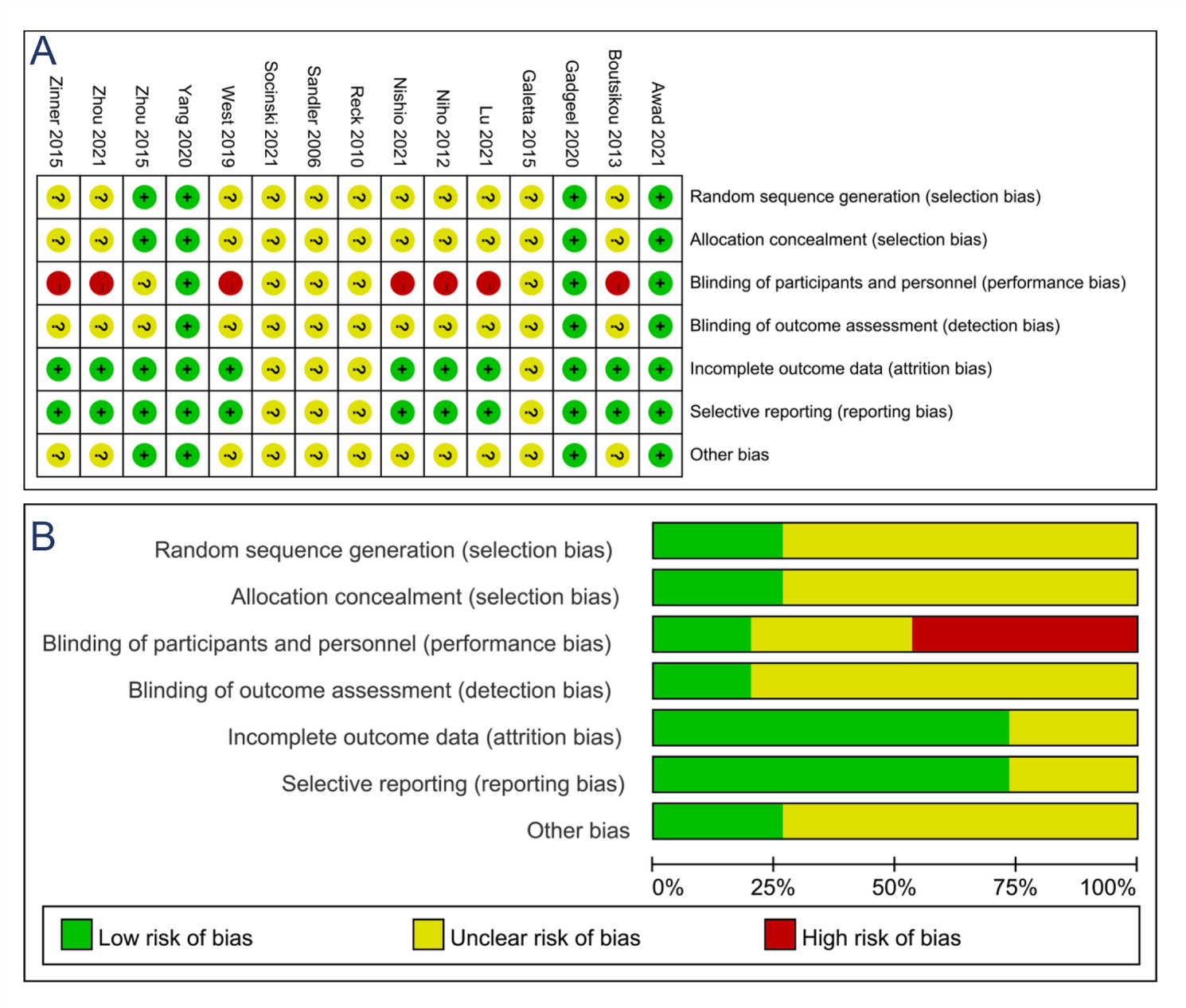


**Supplement 2.** Quality evaluation of included studies. (A) Risk of bias for each included study; (B) Bar chart comparing percentage risk of bias for each included study.

**
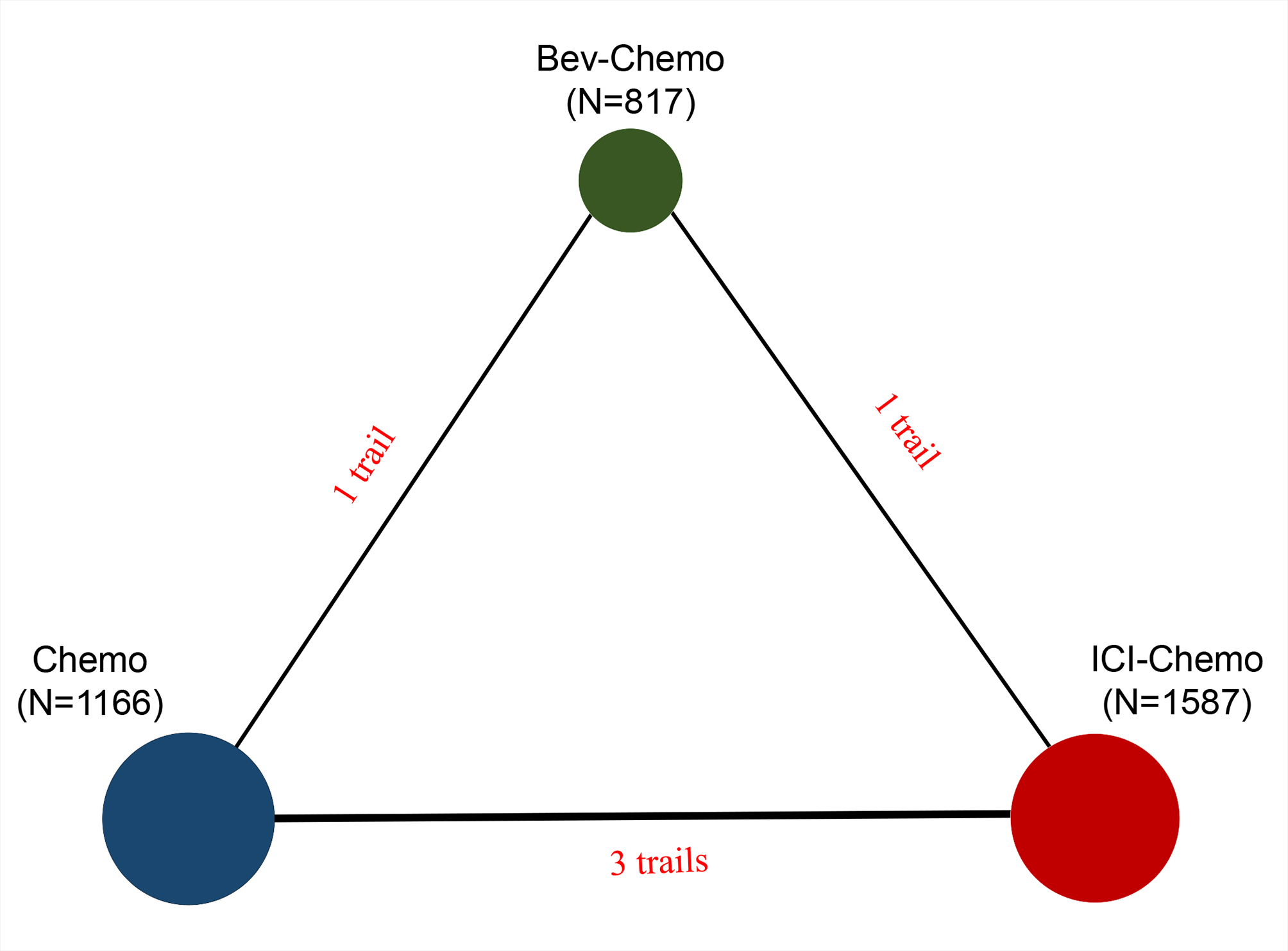
**

**Supplement 3.** Network plot of patients with liver metastases. The area of the circles means the proportional number of patients for each treatment. The thickness of the lines means the proportional number of trials comparing the connected two treatments.

**Supplement 4.** Network meta-analysis of patients with liver metastases

| **HR for OS** | | | **HR for PFS** | | |
| --- | --- | --- | --- | --- | --- |
| ICI-chemo |  |  | ICI-chemo |  |  |
| 1.10 (0.69, 1.80) | Bev-chemo |  | 0.81 (0.29, 2.3) | Bev-chemo |  |
| 0.81 (0.57, 1.20) | 0.73 (0.47, 1.20) | Chemo | 0.66 (0.36, 1.1) | 0.81 (0.24, 2.5) | Chemo |

**
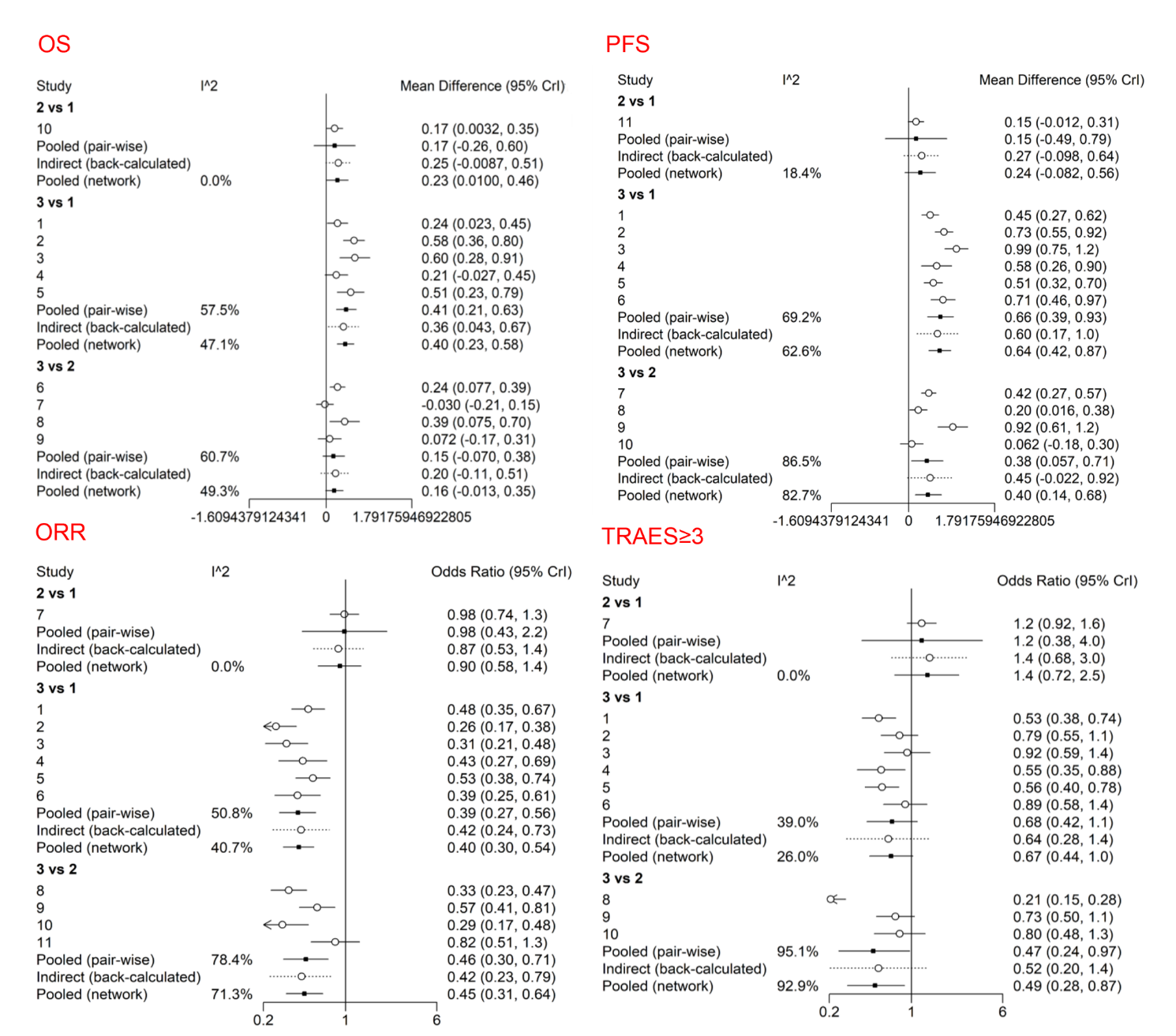
Supplement 5.** Heterogeneity evaluation. Abbreviations: ORR, objective response rate; TRAES, serious adverse events. 1: ICI-chemotherapy; 2: Bev-chemotherapy; 3: chemotherapy

**
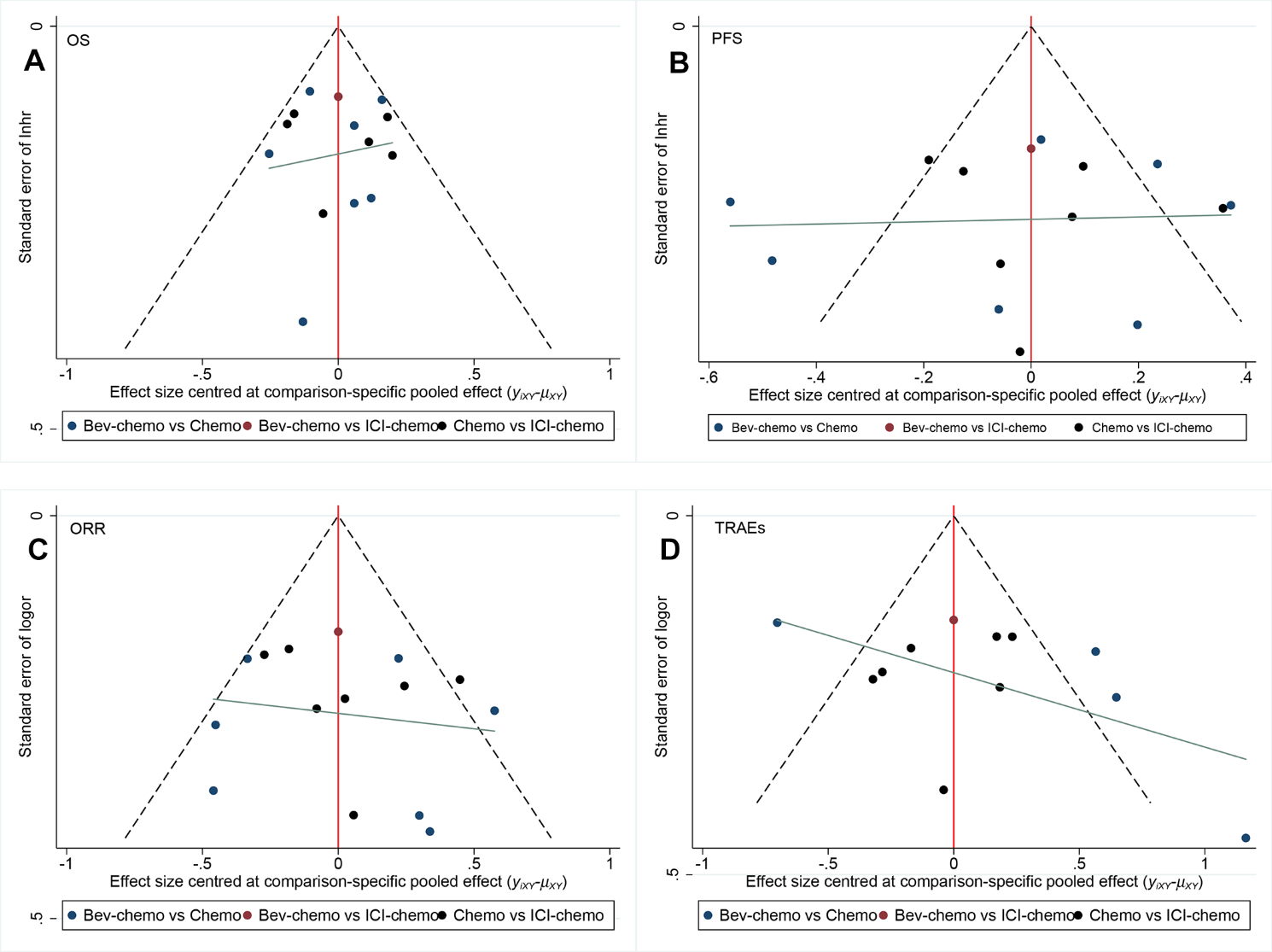
**

**Supplement 6.** Funnel plots for overall survival (A), progression-free survival (B), overall response rate (C), and grade ≥ 3 treatment-related adverse events (D)

**Supplement 7.** Sensitive analysis excluding phase II trials and trials with a sample size of each group below 100

| **HR for OS** | | | **HR for PFS** | | |
| --- | --- | --- | --- | --- | --- |
| ICI-chemo |  |  | ICI-chemo |  |  |
| 0.79(0.63, 0.99) | Bev-chemo |  | 0.78(0.57, 1.10) | Bev-chemo |  |
| 0.67(0.56, 0.79) | 0.85(0.70, 1.00) | Chemo | 0.53(0.42, 0.66) | 0.67(0.51, 0.87) | Chemo |
| **OR for ORR** | | | **OR for TRAEs** ≥**3** | | |
| ICI-chemo |  |  | ICI-chemo |  |  |
| 1.0(0.8, 1.3) | Bev-chemo |  | 0.8(0.6, 1.2) | Bev-chemo |  |
| 1.7(1.4, 2.1) | 1.7(1.4, 2.2) | Chemo | 1.2(0.9, 1.5) | 1.4(1.0, 1.9) | Chemo |

Abbreviation: PFS, progression-free survival; OS, overall survival; ORR, objective response rate; OR, odds ratio; HR, hazard ratio; Bev, bevacizumab; Chemo, chemotherapy; TRAEs, treatment-related adverse events.

**
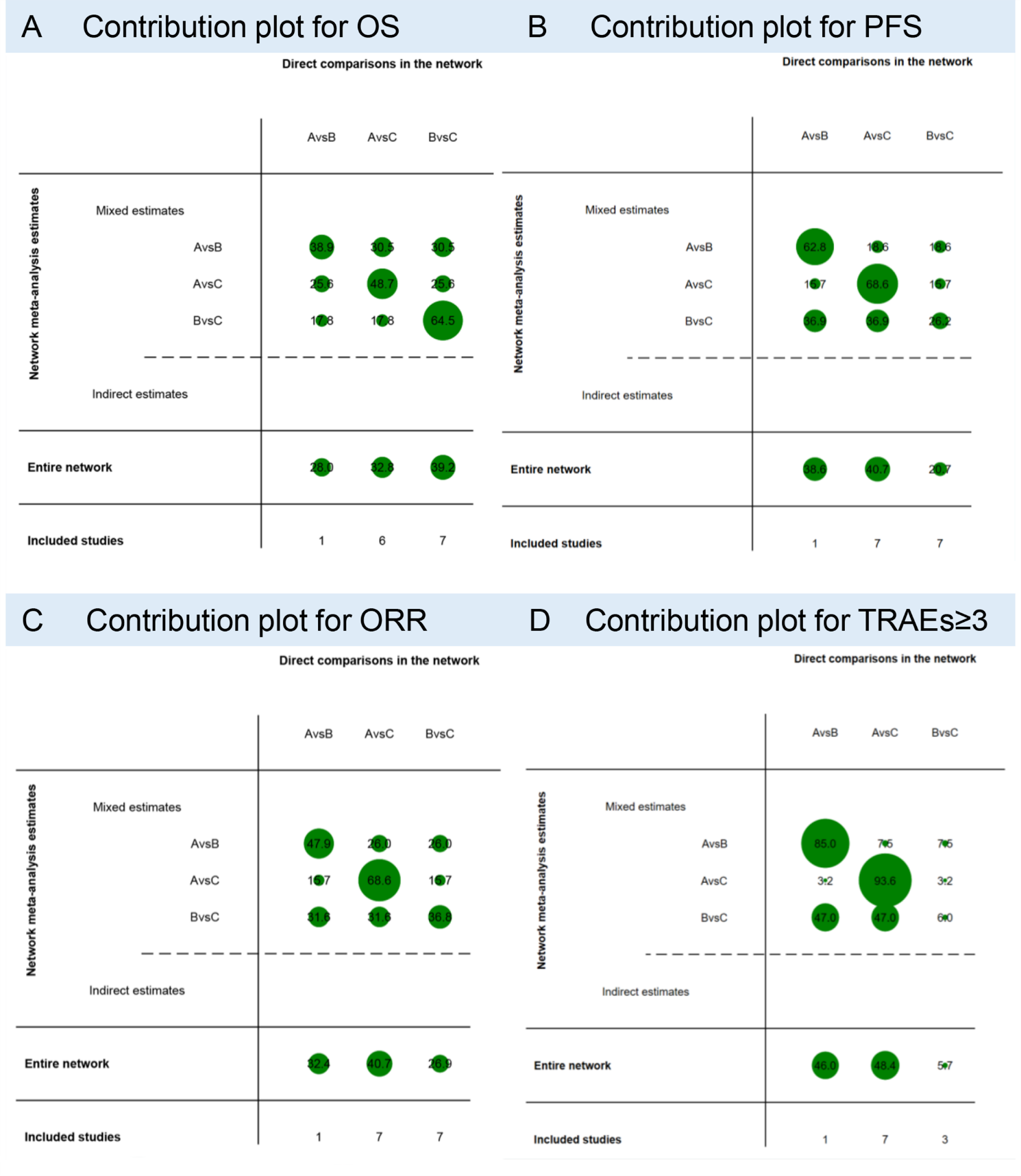
**

**Supplement 8.** Contribution plots for OS, PFS, ORR, and grade ≥3 TRAEs. A: ICI-chemotherapy; B: Bev-chemotherapy; C: Chemotherapy
